# A Virtual Sandbox Approach to Studying the Effect of Augmented Communication on Human-Robot Collaboration

**DOI:** 10.3389/frobt.2021.728961

**Published:** 2021-10-19

**Authors:** Alexander Arntz, Sabrina C. Eimler, H. Ulrich Hoppe

**Affiliations:** ^1^ Institute of Computer Science, University of Applied Sciences Ruhr West, Bottrop, Germany; ^2^ Department of Computer Science and Applied Cognitive Science, University of Duisburg-Essen, Duisburg, Germany

**Keywords:** human-robot collaboration, virtual reality, shared task, augmented communication, production quantity, perceived safety, experimental study, objective measures

## Abstract

Human-Robot Collaboration (HRC) has the potential for a paradigm shift in industrial production by complementing the strengths of industrial robots with human staff. However, exploring these scenarios in physical experimental settings is costly and difficult, e.g., due to safety considerations. We present a virtual reality application that allows the exploration of HRC work arrangements with autonomous robots and their effect on human behavior. Prior experimental studies conducted using this application demonstrated the benefits of augmenting an autonomous robot arm with communication channels on subjective aspects such as perceived stress. Motivated by current safety regulations that hinder HRC to expand its full potential, we explored the effects of the augmented communication on objective measures (collision rate and produced goods) within a virtual sandbox application. Explored through a safe and replicable setup, the goal was to determine whether communication channels that provide guidance and explanation on the robot can help mitigate safety hazards without interfering with the production effectiveness of both parties. This is based on the theoretical foundation that communication channels enable the robot to explain its action, helps the human collaboration partner to comprehend the current state of the shared task better, and react accordingly. Focused on the optimization of production output, reduced collision rate, and increased perception of safety, a between-subjects experimental study with two conditions (augmented communication vs non-augmented) was conducted. The results revealed a statistically significant difference in terms of production quantity output and collisions with the robot, favoring the augmented conditions. Additional statistically significant differences regarding self-reported perceived safety were found. The results of this study provide an entry point for future research regarding the augmentation of industrial robots with communication channels for safety purposes.

## 1 Introduction

State of the art automated production cycles today widely use industrial robots. However, most production processes in heavy industries involve human employees at certain points that either coexist or cooperate with these robots. A shared workspace between humans and robots often demands enormous safety precautions, since robots in these contexts usually possess great physical strength combined with high movement velocities (Meziane et al., 2017). To solve this, strict regulations demand to either fence in these robots or separate them from the workforce. The concept of human-robot collaboration (HRC) requires a paradigm shift for these established safety measures, as this approach envisages industrial robots and employees not only to work together in confined spaces but also to interact directly to accomplish a shared task. HRC creates the potential for new production methods in manufacturing, where tedious, repetitive, and heavy tasks are executed by the robot in collaboration with the adaptive decision-making and individual skill set of the human employee (Ajoudani et al., 2018). Current safety regulations either demand a high expenditure for the collaborative process or diminish the production output (Gerst, 2020). Therefore, jeopardizing the whole concept of HRC, as industries will not invest in complex working arrangements involving collaborative robots that are unprofitable. This requires safety measures, which preserve the individual abilities of both, the human and the robot to contribute to the economic success of the concept through an increase in productivity (Buxbaum et al., 2020). Furthermore, it is anticipated that future iterations of HRC will deploy artificial intelligence, allowing the robot to conduct actions autonomously to some degree. It is assumed that these sophisticated systems will be able to detect their human collaboration partner and act in accordance to prevent hazardous situations (Daugherty and Wilson, 2018). This potential future scenario contains various open questions regarding the design of these working arrangements and people’s reactions towards it (Bröhl et al., 2019).

While prior HRC-related studies explored subjective measurements, the assessment of objective results are also important. As mentioned before, creating a benefit for production output is necessary for the adoption of HRC in the industry, which is partly addressed in the research for creating an effective task execution scheduling aim for the robot (Wilcox and Shah, 2012), experimental studies regarding arrangements with augmented collaborative robots and their influence on productivity and safety still leave space for exploration (Buxbaum and Häusler, 2020). This motivates the aim of this paper to complete the subjective data from our prior studies with objective data that analyzes the effect of augmented communication-based HRC regarding the outcome of production volume and collision rate (Arntz et al., 2020a; Arntz et al., 2020b; Arntz and Eimler, 2020).

Robots that are deployed in HRC industrial scenarios come in many shapes and forms, ranging from robot arms to more obscure appearances such as the Stewart parallel robot (Wen et al., 2018), all designed for a specific required task. Covering all these robot representations for HRC studies provides an enormous challenge, since not every robot nor task is suited to be examined in a lab experiment under controlled conditions. Another crucial factor in experimental studies regarding HRC is safety. Considering that exposing participants to robotic systems with hazardous potential violates any ethical guidelines, thus HRC-related experimental studies conducted with real robots will always be restricted in terms of concepts that can be explored (Liu and Wang, 2020).

In addition to the safety restrictions, the realization of an autonomous collaborating robot requires the usage of sophisticated sensor technology that provides the robot with information regarding its environment (Amara et al., 2020). Prior research circumvented this by using the Wizard-of-Oz approach (Weiss et al., 2009), delegating the control of the robot to the experimental supervisor. Therefore, there is little research that combines an autonomous robot that acts under the guidelines for collaboration along with robots with full interaction exposure within a shared task setup (ISO, 2020).

To address these challenges, we used a virtual reality (VR) sandbox that can be used to create a variety of different HRC scenarios, as the VR technology provides a secure and replicable medium to examine human characteristics when exposed to shared task scenarios involving robots (Matsas et al., 2018). Prior research identified immersion as an essential precondition in the collection of behavioral data through VR that can be projected on the real counterpart scenario (Bailenson, 2018). Since robots in their various appearances and features can be simulated with enough fidelity within the VR sandbox application to match their real counterparts, it can be assumed that the reactions from participants exposed to these virtual robots allow for valid predictions for real HRC setups (de Giorgio et al., 2017). This is backed by the works of Bailenson (2018), who describes the usage of VR technology in a diverse array of social studies, i.e., perspective-taking scenarios where participants assume a different role within an unfamiliar context (Bailey and Bailenson, 2017; Roswell et al., 2020). To provide these contexts within the VR sandbox application, we build a library of prefabs containing the necessary functionality to display a variety of different scenarios, in which any virtual robot arm representation can conduct various actions in conjunction with a human partner. Execution of these actions is based on the implementation of machine-learning driven agents that allow in an innovative way to train the virtual robot arm for various experimental setups and tasks. This enables to design and adjust the behavior of the robot based on the established guidelines and reaction of the participant. Ensuring greater comparability between experimental studies compared to the Wizard-of-Oz approach where nuanced procedural deviations by the human operator can affect the outcome (Schlögl et al., 2013).

In the following sections, we introduce the theoretical background that provides the basis for the formulated hypotheses and the research question. Afterward, the experimental study including the stimulus material is described, in which different augmentation conditions are compared to explore their impact on production quantity and collision rate. Additionally, based on the results of a prior study (Arntz et al., 2020b), we investigate whether the communication augmentations lead to higher perceived safety along with a potential difference in collision rate. Afterward, the results are presented and discussed.

## 2 Theoretical Background

The current theoretical concept of humans collaborating with robots is derived from the group collaboration between human individuals (Shah et al., 2011). Empirical studies in this research field identified group cognition as essential criteria for successful collaboration among humans (Hart and Staveland, 1988). The term group cognition, proposed by Wegner (Wegner et al., 1991), describes a transactive memory system that contains the shared and organized knowledge of a group of collaborating individuals. This organized knowledge contributes to the collaboration performance within a group through a common mental model which is formed through communication (Peltokorpi and Hood, 2019). Depending on the appropriate information suited for the collaboration context that is exchanged through communication this perceived common model can be beneficial. Individuals within a group become more aware of the organization and roles as well as the specific goals of the shared task. The benefit of a perceived common mental model has also been identified in Human-Robot Interaction research, in which the recognition of the robot’s activities combined with a proper reaction to the human commands, can evoke the awareness of group cognition in the human (Shah et al., 2011). This requires a clear understanding of the roles each individual possesses in the process, combined with the prioritization of group needs, which are further aspects for successful collaboration. Applied to the collaboration between humans and robots, the standards are defined as the continuing distribution of sub tasks and immediate coordination of the needed actions to accomplish the common goal (Schmidtler et al., 2014). This requires that the robot must communicate the appropriate proxemics behavior and can follow certain societal norms in terms of gestures and physical contact (Mumm and Mutlu, 2011). However, considering that the majority of robots deployed in industrial environments are built with a non-anthropomorphic appearance (Müller et al., 2017), the formation of such a perception on a cognitive level is much harder to archive than in a robot with a humanoid appearance (Atmaca et al., 2008). Responsible for this are mirror neurons in the brain, which become active while actions are performed by another individual, for the purpose of adapting or improving activities carried out by the respective human (Roesler and Onnasch, 2020). Applied to a collaborative setup, not only the own executed actions, are represented on a cognitive level, but also the anticipation of activities from the partner. A collaboration partner that deviates in its appearance and characteristics, such as an industrial robot can therefore not create the same cognitive stimulus on the human (Sebanz et al., 2005). One approach to induce this stimulus is by eliciting a presence of intention and purposive behavior from the robot through communication (Sebanz and Knoblich, 2009). These characteristics in robots are not only beneficial for the humans’ perception of an intended common goal, the capability for communication also lowers the barrier for perceiving it as a social presence, which can contribute to the willingness of humans to collaborate with it (Heerink et al., 2009). Based on this theoretical foundation the first hypothesis is formulated, which assumes that a robot that is augmented with a communication interface that promotes the aforementioned stimulus, contributes to higher production effectiveness and volume within a shared task setup. Although contributions for increasing productivity through HRC are the largest advocates for establishing the concept of collaborating with autonomous robots in the industry, the research focused on these aspects is still in its infancy and should be explored more (Galin and Meshcheryakov, 2020), as comparable studies omit the augmentation aspect of the robot (Heydaryan et al., 2018).

Apart from productivity, another concern for the industry regarding HRC is safety. Currently, potential hazards from the robots are diminished by dividing HRC into three categories: In the first, employees are shielded from the robot either through cages or separated working areas (Haag, 2015). This enables the robot to work faster as no precautions are needed to take for avoiding trespassing human workers. The second category restricts access to the robot. A designated area that separates the robot from its co-workers is omitted, instead, sensors form a light curtain around the robot (Haag, 2015). If the curtain is breached, the robot ceases its current motion. Due to regulations (Rosenstrauch and Kruger, 2017), demanding a generous safety radius around the robot, no direct interactions between the robot and the worker are allowed. The third category uses proximity sensors to calculate the distance of the worker to the robot (Haag, 2015). With these categories designed to meet current technical limitations, the introduction of AI-based robots in shared tasks (Lenz and Knoll, 2014) will likely enable the detection of the motion of intervening employees and to anticipate the movement of people and objects (Zakka et al., 2019). Same with conveying the robot’s actions, communicating the detection of potential collisions and their influence in reducing potential accidents are questions of interest regarding HRC (Buxbaum et al., 2020), which will be investigated in the second hypothesis.

The anticipated decrease in collisions enabled by the communication channels is also expected to increase the perception of safety within the collaboration task. This can be attributed to the contribution of communication between entities to the perception of safety within a workspace (Seo, 2005). The safety of a workplace is influenced by a variety of dimensions and can affect the safety performance and perceived safety of an individual (Fernández-Muñiz et al., 2012). One of the frequently discussed dimensions is the awareness of the organizational structure of a task, which in the case of collaborative work is directly linked to the exchange of information regarding the task management (Cigularov et al., 2010). This led to the formation of the third hypothesis, as the communication channels of the robot could raise people’s perception of safety in the system compared to a robot without augmented communication capabilities. In addition to the formulated hypotheses, the time participants gazed onto the guidance and explanation provided by the text panel channel was of interest, resulting in the research question investigating whether the time affects the productivity of the participants or the collision rate with the robot arm.

### 2.1 Hypotheses

For the purpose of exploring the effect on production capacity, collision avoidance, and the perceived security of guiding and explanatory augmentation of industrial robots in shared task environments, the following hypotheses were formulated:

• H1: Participants produce more pin-back buttons in the augmented condition compared to the non-augmented condition.
• H2: Participants collide less with the augmented robot arm compared to the non-augmented condition.
• H3: Perception of safety is higher in the augmented condition compared to the non-augmented condition.
• Research question: Does the time participants look at the text panel affect the productivity and collision rate?

## 3 Experimental Study

### 3.1 Methods

The experimental setup varied the presence vs. absence of augmented communication channels in a between-subjects design where participants were tasked to assemble pin-back button components in collaboration with the autonomously acting robot arm in VR. In the experimental condition, the robot arm was augmented with the three aforementioned communication channels. The non-augmented condition omitted these communication channels.

The sample size was *N* = 80 (40 female), with 40 participants assigned to each of the two conditions. Both conditions contained an equal gender distribution. The average age of the participants was 25 (*M* = 25.31, *SD* = 6.10). The majority of the participants were students with a background in computer science and engineering from the University of Applied Sciences Ruhr West.

### 3.2 Stimulus Material

**FIGURE 1:**
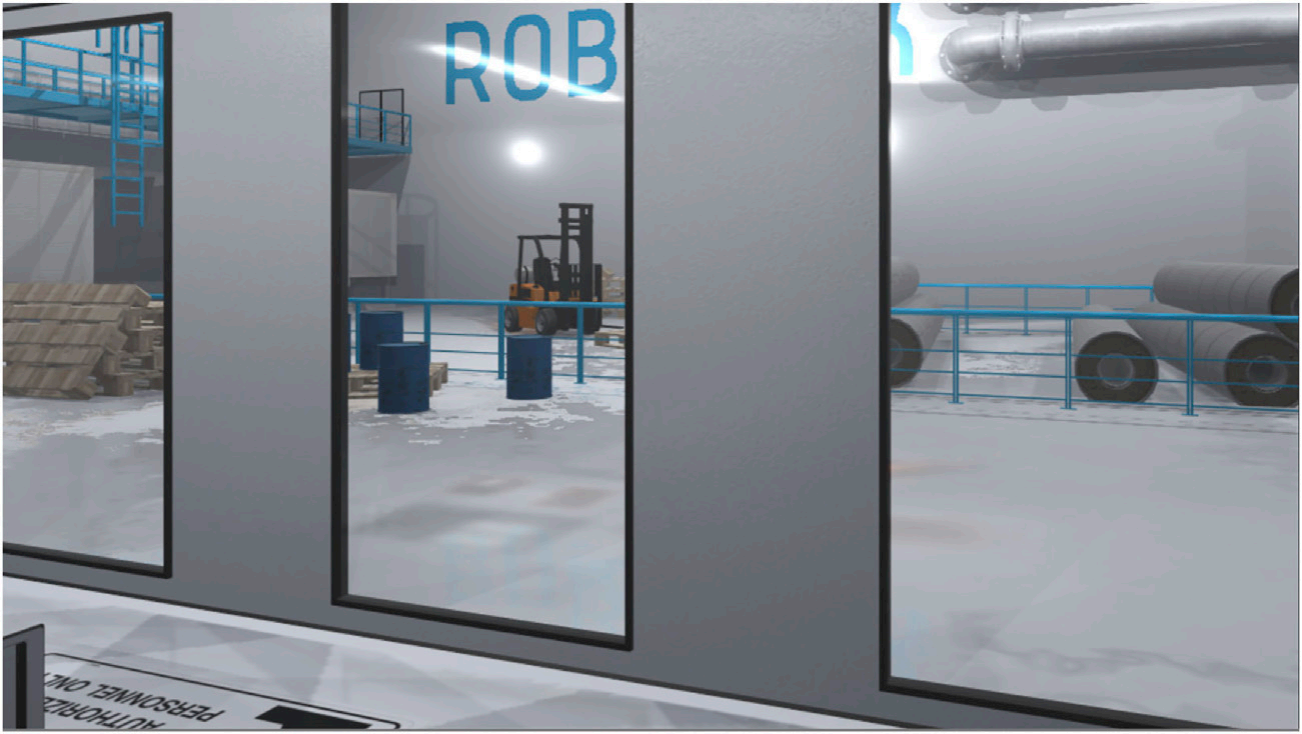
The virtual representation of an industry environment served as the background of the workplace arrangement. Equipped with appropriate props and sound cues, this backdrop aimed to provide the context of the setting and facilitate the immersion of the experimental study.

To facilitate immersion in the VR sandbox experiments, a virtual environment that emulates an industrial workspace was required. To ensure an authentic depiction, four industry representatives and robot experts were involved in the design process. Qualitative interviews conducted with the experts helped to identify appropriate machinery used in manufacturing plants, the layout of common HRC working arrangements, frequent procedures, and the design of the communication channels. Additional reference material complemented the remarks stated in the interviews (Vysocky and Novak, 2016; Villani et al., 2018), resulting in the final creation of the virtual environment implemented in Unity 3D (Version 2018.4.11f1) (Unity, 2020a) (Figure 1).

To ensure stable performance of the virtual environment despite being filled with a variety of props, i.e., pipes, forklifts, and cables. Streaming assets and shader of the objects were optimized for VR usage. This ensured reaching a target rate above ninety frames per second which is crucial for virtual reality, reducing side effects such as motion sickness or eye strain (Jerald, 2016). Non-interactive assets were placed as static objects into the scenery, which allowed for a mixed lighting setup with baked shadow maps for immovable objects and real-time lighting for interactive and dynamic objects. This, in conjunction with the use of pre-calculated reflection cube maps, allowed for a much more elaborated visual fidelity adding to the immersion. The ambient soundscape completed the experience with various industrial background noises composed of public domain audio files mixed with recordings from a steel mill, taken from a preceding project (Zengeler et al., 2020).

**FIGURE 2:**
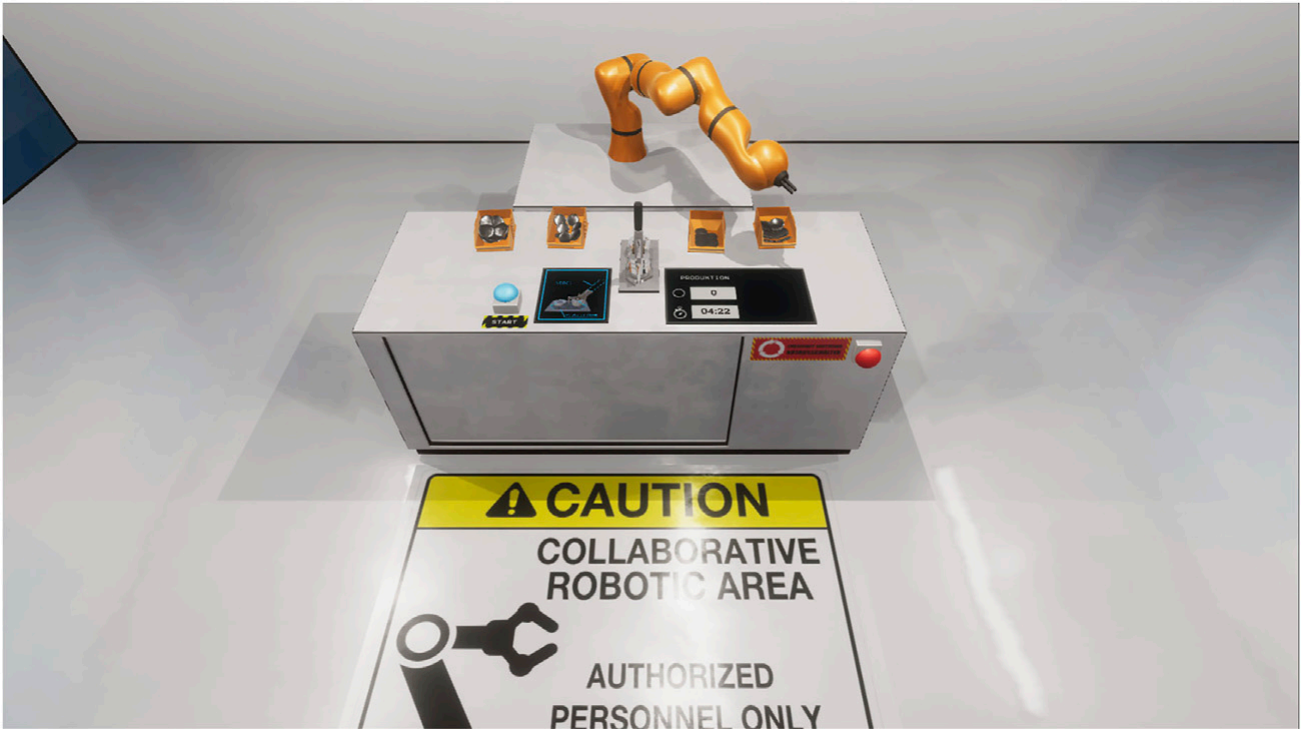
The virtual workplace arrangement at which the participants conducted the shared task in collaboration with the robot arm. Apart from the pin-back button press and the container for the assembly components, the workplace contained a start and emergency shutdown button. Shown is the non-augmented condition where the three communication channels are absent. The virtual workspace where the shared task was executed by the participants in collaboration with the robot arm. The arrangement was designed Note that in the control condition the augmented communication channels are absent.

The locomotion mechanic was implemented through the Oculus API and allowed the users to ambulate either through the controller or by their natural body movement. To discourage the exploration of the environment and keep participants focused on the goal of the HRC workplace, the arrangement was enclosed in a separate room that provided a barrier without breaking the internal consistency of the virtual environment. The workplace arrangement itself consisted of a waist-high desk, where the shared task can be executed in collaboration with the autonomous robot arm (Figure 2).

#### 3.2.1 Shared Task

The VR sandbox is designed to address numerous categories of collaboration tasks and procedures. For this purpose, a diverse array of interaction mechanics were implemented that allows manipulating actions such as pushing and pulling virtual objects. The usability of the actions was designed according to established third-party applications like the virtual reality toolkit (VRTK, 2020). For the context of the designed experimental setup, it was necessary to provide a shared task that included the participation of both parties in assigned roles following a coherent representation of a manufacturing process.

Investigating several manufacturing processes involving the usage of collaborative robots in real industries, resulted in cumbersome procedures that were deemed too taxing for inexperienced participants. For this purpose, a comprehensible alternative was conceived in the usage of the Badgematic Flexi Type 900 (59 mm) press as a shared task to produce pin-back buttons (Badgematic, 2020). The use of stand-ins for real manufacturing tasks can be found in several research setups involving HRC (Sen et al., 2020; Williams et al., 2020).

**FIGURE 3:**
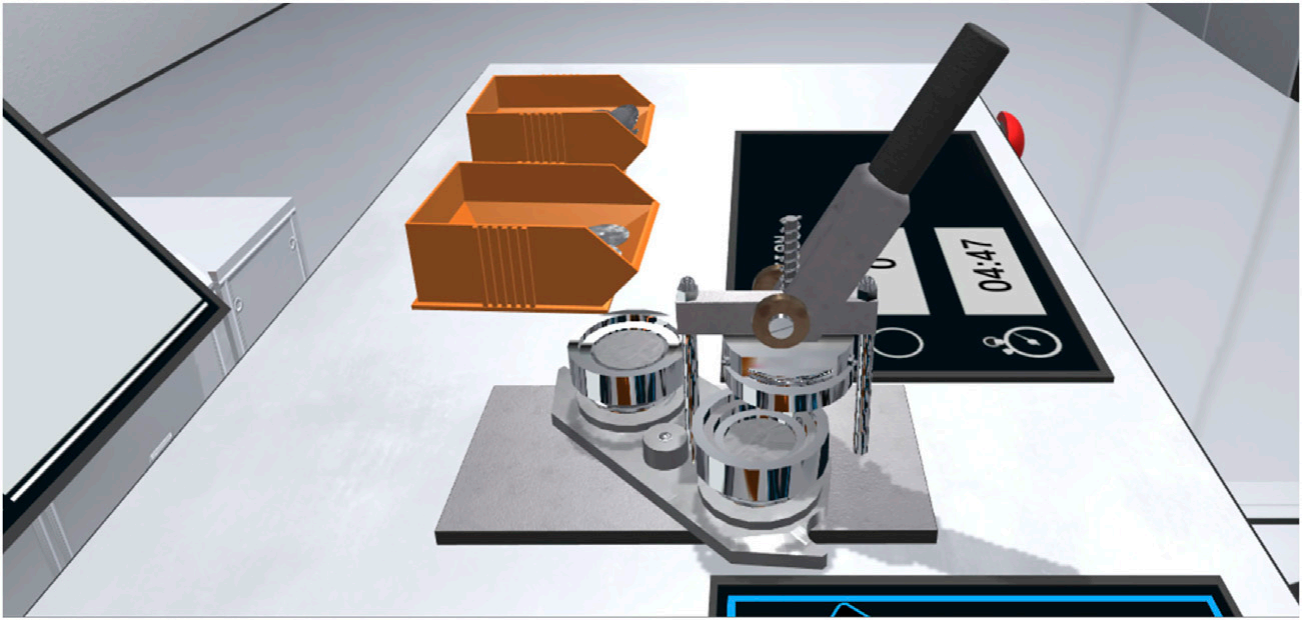
The Participants operated the virtual pin-back button press in collaboration with the robot arm. The implemented interaction mechanics emulated the physical button press and enabled participants to use the lever and the rotation tray. Authentic sound and haptic feedback completed the representation.

The virtual representation of the button press was authentically modeled after the real one, using Autodesk Maya 2018 (Figure 3) (Autodesk, 2020). The pin-back button press consisted of three components. While the frame of the press itself was static, the stamp platform of the press and the associated lever were intractable by the participants through the usage of the Oculus Rift touch controller. To mimic the real characteristics of the button press, both interactive components were equipped with a hinge point and a rotator that interacted with the handgrip mechanic of the Oculus integration (Oculus, 2020b). Simulated friction was implemented to create the illusion of a resistance that is required when using the lever or turning the stamp platform. Audio sources were added to the components of the pin-back button press, which emitted sounds recorded from its real counterpart, varying in intensity based on the force of which the lever is pulled, the stamp platform is turned or a segment of the pin-back buttons is either inserted or extracted.

**FIGURE 4:**
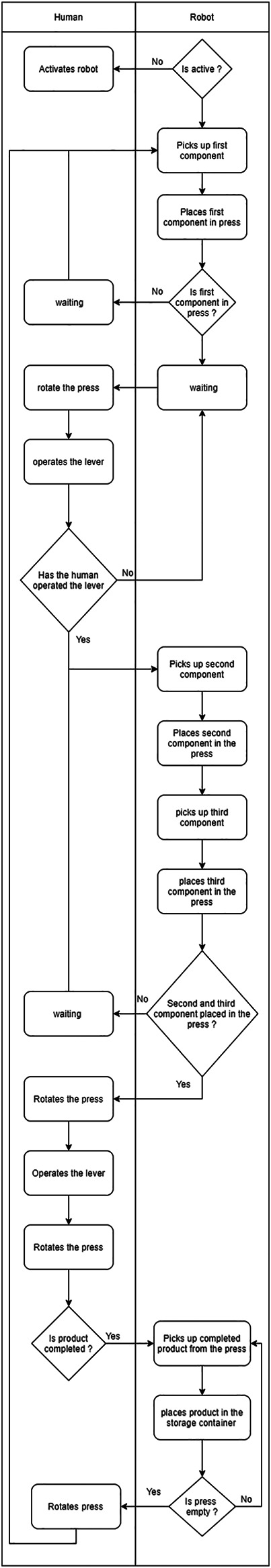
The diagram illustrates the individual procedures required by the human participant and the virtual robot arm to execute the shared task.

The shared task itself involved a total of nine individual working steps which were executed alternately between the human participant and the autonomous robot arm (Arntz et al., 2020a) (Figure 4). The procedure was initiated by the participant pressing the start button. The robot arm then moved to the respective storage container to pick up the first component of the pin-back button. After the robot arm grabbed the first component, it was inserted by the robot arm into the first tray of the pin-back button press. The robot arm retracted then to make way for the participant, who was required to rotate the press tray and operate the lever of the pin-back button press. The next step was for the robot arm to transfer the second and third pin-back button component successively into the empty remaining tray. Subsequently, the press tray was again rotated by the participant followed by pulling the lever and another press rotation. The robot arm was then tasked to extract the finished pin-back button from the press and move it to the respective storage container. Once a full production cycle was complete, the process for the production of the next pin-back button began immediately. The number of the produced pin-back buttons in conjunction with the remaining time was displayed to the user via a virtual monitor placed on the work desk in front of the participant. An emergency shutdown button that terminated all operations from the robot arm gave participants additional security measures and was designed and implemented following common industry safety protocols (Heydaryan et al., 2018).

#### 3.2.2 The Collaborative Robot Arm

Although the VR sandbox was created to allow any form of robot collaboration partner to be evaluated, this scenario used a representation of the KUKA LBR iiwa 7 R800 CR series (KUKA, 2020), which is widely used in various industries and application scenarios. To ensure an authentic portrayal of the virtual robot arm, reference manuals and schematics from the manufacturer were consulted in combination with intensive examination of the real pendant (Kresse, 2010; Lemaignan et al., 2014; KUKA, 2016; MORSE, 2020). Also of importance was the sound for auditory location in collaboration setups involving robots (Cha et al., 2018). Multiple sound recordings from the real robot arm were combined to recreate the distinctive soundscape of the LBR iiwa series through the audio tools of the Unity 3D engine. This resulted in an accurate representation of the visuals and characteristics of the robot arm.

The collaboration aspect of the experimental setups within the VR sandbox demanded the robot arm to react adequately towards the actions of the participants. Therefore, the usage of an animation controller that contains a pre-defined set of animated movements was rejected in favor of an inverse kinematic system. This allowed calculation of the required joint angles for the robot arm to reach any target position as well as dynamic movement. Following the structure of the real LBR iiwa series, the virtual model comprised seven degrees-of-freedom (DoF) in a spherical-rotation-spherical kinematic structure using the same parameter as the real robot arm (Faria et al., 2018; Doliwa, 2020a). The inverse kinematic implementation for the VR sandbox was based on a closed-form solution, which provided better performance compared to a numeric solution (Artemiadis, 2013). The inverse kinematic system made use of the Denavit-Hartenberg parameter, as the basis for the calculation in 7-DoF (Faria et al., 2018). In addition to the movement characteristics, the range of angles, the joints can cover derived from the real LBR iiwa series had to be implemented to prevent that the robot arm moves through itself (Doliwa, 2020b). For further interactions with the environment and the participant, each segment of the robot arm was outfitted with collision properties using the Unity 3D built-in tools enabling it to register contact with other objects. This also allowed to monitor and record the robot arms collision rate for the objective data acquisition.

#### 3.2.3 Capabilities of the Robot Arm

To present a wide range of collaboration setups with autonomous robots via the VR sandbox, it was necessary to implement the ability of the robotic arm to perform the collaborative task independently of an external controller such as the Wizard-of-Oz approach. Although the working steps for the robot arm to execute within most collaboration tasks are determined, the actions of the human collaboration partner introduce an unpredictable element, to which the robot arm must react adequately in a functional, predictable or legible way (Dragan et al., 2013). For the VR sandbox, the capabilities of the robot arm were implemented based on the following goals:

• Identification: the robot arm is required to detect the movement of the participant represented by the hands and the head of the VR avatar and takes countermeasures to avoid dangerous collisions.
• Adaption: The robot arm should adapt to the work pace of the participant and either increase or decrease its movement speed in accordance with the ISO TS 15066 regulations.
• Execution: The robot arm can complete its working part of the shared task.
• Verification: the robot arm is capable of recognizing that the action of the participant follows the working procedure
• Notification: the robot arm is capable to communicate its actions and possible detected deviations from the procedure.

**FIGURE 5:**
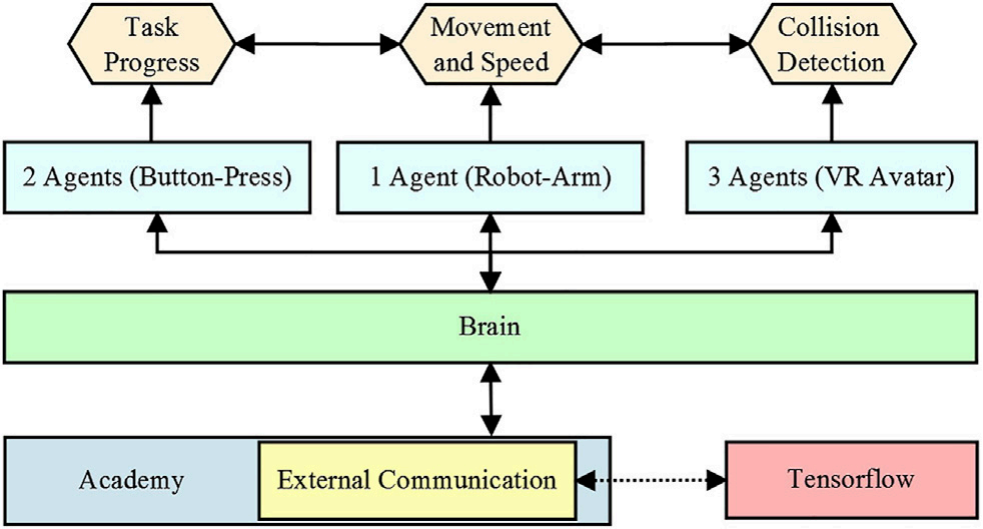
The schematics of the ML-Agents framework consisted of three components, the agents, the brain, and the academy (Juliani et al., 2018). The agents monitored the states of the environment and assumed control over the robot arm. Determined by the brain, the robot arm acted based on learned behavior stored in the academy.

Incorporating the Unity 3D Machine Learning Agents (ML-Agents) in conjunction with the inverse kinematics system enabled the robot arm to conduct these defined characteristics. The ML-Agents open-source plugin provides a framework for the application of various machine learning methods, i.e., reinforcement learning to virtual objects through a Python API and the TensorFlow interface. The ML-Agents SDK itself contains three major components: The first is the agent, which gathers information about the current state of the scene and can execute actions. These actions are determined within the second component, the Brain, which contains the various rules and conditions for the decision-making of each of the corresponding agents. The third component is the Academy, responsible for the global coordination of the simulated environment (Juliani et al., 2018) (Figure 5).

For the ML-Agents framework to assume control over the inverse kinematic system of the robot arm, a Unity GameObject serving as the target for the inverse kinematics-solver was equipped with the provided agent component from the SDK. This agent determined the movement and the speed at which the robot arm heading for its target. The procedure of the shared task with its designated roles was then modeled by adopting the Relational Action Processes (RAP) established by Toussaint et al. (2016). Through the usage of the relational Markov Decision Process, which is commonly implemented for decision processes of agents performing within an environment, the model enabled the simultaneous operation of several actions, either sequential or asynchronously, depending on the current requirement (Munzer et al., 2018). Additional information from two agents monitoring the states of the pin-back button press tracked the current progress of the task and the speed at which the participant conducted it, were used to enable the robot arm to adapt its movement speed to the working pace of the human partner. This increase in speed was limited by the ISO TS 15,066 regulations (ISO, 2020). Further information regarding the movement of the robot arm was relayed from three agents attached to both hands and the head of the VR avatar, for the robot arm to avoid collisions with the participant. Depending on the current speed the robot arm either attempted to evade the participant while slowing down incrementally or ceasing all motions instantly. This was implemented mimicking the real characteristics of the real robot arm model, as the robot arm has to intercept its momentum, therefore a certain breaking distance is required.

Also, the possibility that the robot arm could be stuck either by the surrounding objects or by a loop had to be considered and counteracted. For this purpose, Unity’s built-in collision system was complemented by a raycast system that sends out radial rays to detect surface meshes of the 3D objects in the vicinity, as the existing Unity collision system only detects entering and exiting collision states. Conducting the learning process of the robot arm without the raycast system would distort the outcome as the ML-Agent framework would not notice states of continuous collision from the robot arm with adjacent objects. A reward system for following the current required target while considering the state of the other agents monitoring the various other items within the virtual environment and punishment for moving away was implemented. Based on the different states of the items necessary for the shared task, these rewards and punishments were adjusted or inverted, enabling the robot arm to follow the procedure for producing a pin-back button in collaboration with human input in this experimental setup (Arntz et al., 2020a). This allowed the robot arm to react and adjust to user input and follow the necessary working procedure while adapting its operating speed over time to keep pace with the participant.

The agents were trained by using recorded data from the collaboration process within the application from nine sessions conducted with three individuals each. A single training segment was defined as the necessary actions for the agents to accomplish the individual working steps of the procedure. The segment was considered to have failed, if the agents reached a collision score of fifty in conjunction with more than eight hundred attempts to reach the respective target, i.e., removing the pin-back component from the press.

An expected disadvantage of an agent-controlled kinematic system compared to predefined animations is the potential tremble in the movement due to noise in the training sample. To mitigate this, a per degree movement penalty was implemented to smooth out the motion of each joint of the robot arm as much as possible, ensuring a close depiction of the virtual robot arm’s movement to its real counterpart.

An interface component managed the transfer of variables between the agents and the scripts attached to the various non-interactable objects within the environments, such as the display that presented the production quantity to the respective participant. The same approach was used for the three distinct augmentation channels for the communication methods.

#### 3.2.4 Augmenting Channels for Guidance and Explanation

**FIGURE 6:**
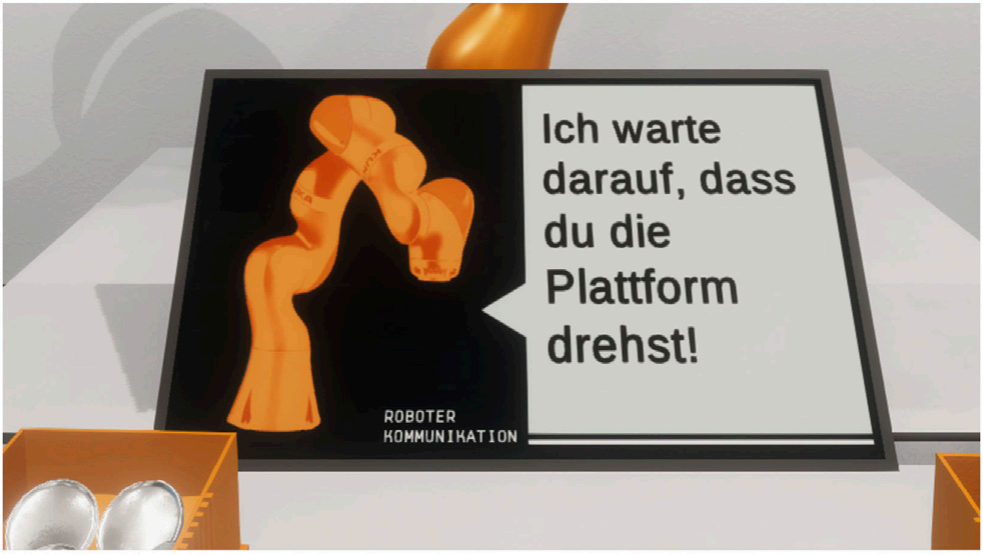
The text panel provided guidance for the current task and an explanation of the robot arms’ behavior. The text was displayed within a speech bubble next to a stylized representation of the robot arm to strengthen the affiliation of the statements to the robot arm. The communication was formulated in the first-person form to evoke the perception of the robot arm as a collaboration partner instead of a tool (“*I’m waiting for you to turn the platform*”).

To evoke the impression of an intended behavior, three distinct unidirectional communication channels were conceptualized. Based on a pre-study (Arntz and Eimler, 2020), the following augmentations were implemented for the VR sandbox: 1) Text communication in natural language, 2) Multi-colored light signals, 3) Action initiating/terminating and standby gestures. The essential purpose of these augmentation channels was to notify about the progress within the task procedure, explain the current action that the robot arm conducted, alert any potentially hazardous situations, and provide feedback to the activities of the human collaboration partner. The first goal was realized through the text communication panel, which was represented through a virtual display containing written statements that explained the ongoing action of the robot arm. To enhance the associations of these statements to the robot arm, the virtual display was placed directly in front of the robot (Figure 6). A pre-study revealed that the adjacent positioning of the virtual display strengthened the impression that these statements originated from the robot arm (Arntz and Eimler, 2020). This was complemented through a stylized graphic of the robot arm that was placed right next to the text, which was embedded in a speech bubble. The text itself was formulated in the first-person form to give a further impression of an intended behavior, a design choice taken from voice assistants, such as Amazon Alexa and Apple Siri (Hoy, 2018). Although the phrasing of the statements from the text panel emulated a personality akin to the aforementioned voice assistants, the usage of speech by the robot arm was dismissed for this experimental study. Several qualitative statements from the prior study indicated that the presence of voice output encouraged the user’s expectation of voice input (Arntz and Eimler, 2020). Since many available conversational AI and natural language processing tools are designed to recognize speech patterns in soundscapes polluted through the presence of other media devices (Papayiannis et al., 2018), no robust solution for industrial ambient noise was available. Although the text panel denies the capability for two-way communication exchange, it was suitable for the intended goal of this study to provide explanation and guidance. In total, the robot arm was able to express forty-two pre-defined statements, counting three variations for fourteen distinct statements to avoid sequential repetitions of the phrasing. To implement the text communication channel, a Unity UI (user interface) Canvas was placed in the world view of the scene which contained a label element. The text was then displayed through Unity’s build-in text rendering technology TextMeshPro with no additional performance cost (Unity, 2020b).

**FIGURE 7:**
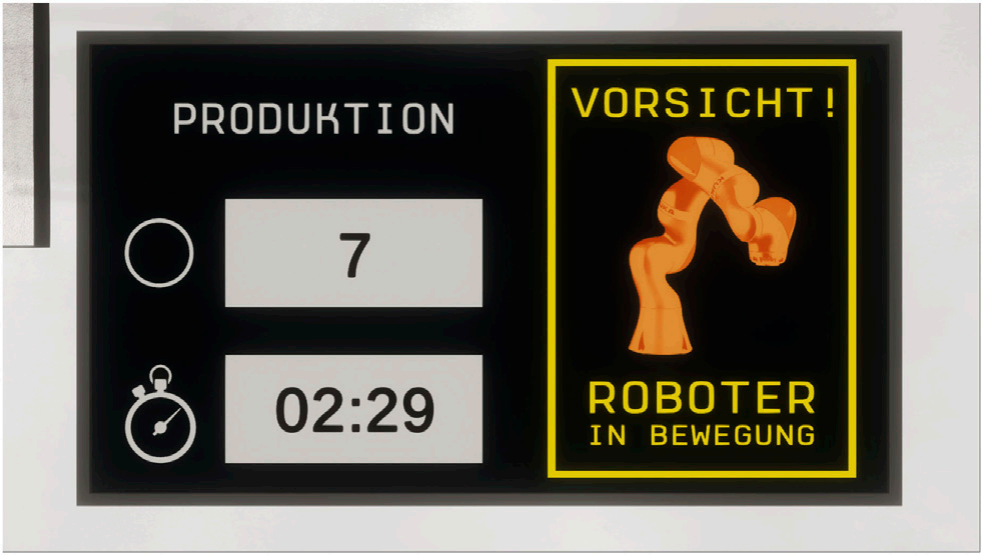
Additional notifications complemented the communication channels, informing the participant about the current activity status of the robot arm (“*Caution! Robot in motion*”). The left side of the display contained information about the shared task by showing the remaining time and the production quantity to the participant.

The second augmentation consisted of multi-colored light signals, which were directly attached to the actuators of the robot arm. The concept of these light signals was to alert for potentially dangerous situations with a visual stimulus that is directly in the field of view of the participant and comprehensible at a glance. Derived from suggestions made from qualitative statements from a preceding study (Arntz and Eimler, 2020), a green light was used for signaling the normal operation of the shared task, while a red light indicated erroneous deviance from the procedure or a detected collision. The light signals were implemented by using a light-emitting shader on the actuator rings of the robot arm model. Based on the received input, the shader changed its color properties and was able to switch from red to green and vice versa or black in the case the robot arm was shut down. To provide further illumination of the surroundings, points lights were attached to the light signals to enhance the visual fidelity. To add a further explanation, the light signals were accompanied by notification labels that were shown on a virtual display (Figure 7). A green light signal was shown in conjunction with a general caution warning, that reminded the participant that the robot arm was in motion. If the red light signal was triggered based on an imminent collision, a warning label alerted the participant that he/she was too close to the robot arm.

The third augmentation was the capability of the robot arm to conduct three gestures (action initiating, action terminating, and standby). Apart from the general approach of providing guidance and explanation, the capability of using gestures was implemented to strengthen the perception of an intended behavior from the robot arm and contribute to the safety attribution of the system. The purpose of the action initiating gesture was to signal the human to proceed with the objective in case no action by the participant was detected. If the collaboration process was stalled through the participant’s inactivity, the robot arm pointed towards the object that was necessary for the subsequent working step (Arntz et al., 2020a). The concept behind this gesture was to reinforce the impression of agency by the robot arm to pursue the objective of the shared task. The counterpart was the action terminating gesture, that was triggered if deviance from the procedure was detected. The robot arm erected its front and rotated the front section with the attached clamps similar to a dismissive hand wave (Arntz et al., 2020a). The goal was not only to notify the human collaboration partner of an incorrect action but also to evoke the impression that the robot arm has a sense of awareness. The same applied to the standby gesture, where the robot arm retracted itself from the button press after completing its working step (Arntz et al., 2020a). This was implemented to enable the robot arm to make room for the human collaboration partner to conduct their activities and meet the expectation of the appropriate proxemics (Mumm and Mutlu, 2011). The design of these gestures was inspired by Ende et al. (2011), who evaluated several approaches for gestures in collaborative working processes. To further enhance the perception of safety the works of Koay were consulted, regarding the movement of the robot arm (Koay et al., 2017). The behavior of the robot arm was adapted to consider social norms for personal space and avoiding sudden motions that could be interpreted as threatening by some people.

### 3.3 Measures

To measure the number of produced pin-back buttons and the collision rate, objective data tracked by the VR application were used. The designated data-set for productivity measured the quantity of pin-back buttons the participant produced in collaboration with the robot arm (H1). The second objective data set detected the number of collisions the participant had with the robot arm (H2). A third objective measure tracked the duration in seconds the participants watched the text panel with the guidance and explanation provided by the augmented robot arm. This measure was only present in the experimental condition, due to the absence of the text panel augmentation in the non-augmented condition.

In addition to the objective measurements, self-reported data were surveyed. The used questionnaire was formulated in German. Items either taken or altered from sources in the English language were translated to German by one researcher and then translated back independently by another researcher to ensure correctness. Measuring the influence of the robot arm’s augmentation on the perceived safety of the participants was done by utilizing self-reported questionnaire data. To measure the perception of safety provided by the augmentation channels of the robot arm (H3), four scales were used. The first contained five items regarding safety aspects of the workplace (*α* = 0.69; Table 1) measured on a 5-point Likert scale (1 = very dissatisfied; 5 = very satisfied) which were modified by adding the word virtual to fit the context of the experimental setup from the Construct validity of a physical work environment satisfaction questionnaire (Carlopio, 1996). The second scale covered the perceived safety of the robot arm with three items (*α* = 0.66; Table 2), measured on a 5-point Likert scale (1 = strongly disagree; 5 = strongly agree) based on the survey methods for Human-Robot Interaction established by Lasota et al. (2017). The survey contained four items of the perceived safety scale. One of which was excluded because it negatively affected the reliability. The fact that Cronbach’s alpha value is below 0.7 can be explained by the small item size of the used scale (Bujang et al., 2018). While a low alpha is generally considered unfavorable, according to George and Mallery and supported by Hinton et al., an alpha value between 0.6 and 0.7 is still valid for statistical operations (Darren and Mallery, 2003; Hinton et al., 2014). The third scale rated the augmentation channels of the robot arm in terms of comprehensibility and effectiveness (4 items, *α* = 0.85; Table 3) measured on a 5-point Likert scale (1 = very bad; 5 = very good). General satisfaction regarding the collaboration with the robot arm was assessed by using four items rated on a 5-point Likert scale (1 = very satisfied; 5 = very unsatisfied) (*α* = 0.72; Table 4). Furthermore, the pre and post-questionnaires contained various items. i.e., regarding the assessment of the robot arm in terms of prior experience with industrial robots and the second edition of the Technology Acceptance Model (TAM2) which were used as control variables (Arntz et al., 2020a).

**TABLE 1:** Workplace safety is measured by the items derived from the physical work environment satisfaction questionnaire by Carlopio (1996).

For each statement, please consider to what extent you think it is true	
No.	1 = very dissatisfied; 5 = very satisfied
1	How satisfied were you with the security measures in your virtual workspace?
2	How satisfied were you with the overall design of your virtual workspace?
3	How satisfied were you with the amount of time the robot gave you to do your work?
4	How satisfied were you with the amount of work you needed to complete the task?
5	How satisfied were you with the amount of work the robot required to complete its task?

**TABLE 2:** The items for the perceived safety scale are based on the scale by Lasota et al. (2017).

For each statement, please consider to what extent you think it is true	
No.	1 = strongly disagree; 5 = strongly agree
1	I am of the opinion that an accident with the robot (e.g. a collision) can happen or can happen again
2	I felt safe in the presence of the robot
3	I believe that other people feel safe in the presence of the robot

**TABLE 3:** The items used for the rating of the communication channels.

For each statement, please consider to what extent you think it is true	
No.	1 = very bad; 5 = very good
1	In general, the robot’s communication was…
2	The robot’s light signals were…
3	The robot’s text panel cues were…
4	The robot’s gestures were…

**TABLE 4:** The items measuring the satisfaction regarding the collaboration with the robot arm.

For each statement, please consider to what extent you think it is true	
No.	1 = very dissatisfied; 5 = very satisfied
1	How satisfied were you with the efficiency of the robot?
2	How satisfied were you with the robot’s effectiveness?
3	How satisfied were you with the flexible working speed of the robot?
4	How satisfied were you with the danger warnings you received from the robot?
5	How satisfied were you with the way the robot tries to avoid accidents?

### 3.4 Experimental Procedure

At the beginning of the experimental study, participants were asked to sign a declaration of consent. This was followed by a short briefing, informing the participant about the aim of the study. Subsequently, the participants were asked by the study supervisor to complete the pre-questionnaire, provided through a desktop computer present in the lab. A small wall gave the participants the privacy to answer the pre-questionnaire without time constraints.

The next stage was the use of the VR application. The supervisor instructed the participants about the Oculus Rift S VR hardware (Oculus, 2020a), its usage, and controls. With no questions remaining, the participants were provided with a special disposable mask, to enhance hygiene and reduce wear on the device. The VR headset was properly mounted, a tutorial scene was loaded. This scene contained the full industrial environment, without the robot arm. The purpose of this was to allow participants to get used to the VR experience and the interaction mechanics of the virtual environment. With about fifteen square meters of free-range, participants were provided with enough space to move within the restrictions of the connection cable of the device. Once the participant has signaled to be ready, the actual stimulus material was loaded, containing the shared task environment with the autonomous robot arm. After the collaboration process started, the participant was given 10 min to produce as many pin-back buttons as possible, following the procedure described in Section 3.2.1. After the remaining time had been up, the application informed the participant that the procedure has ended.

The supervisor aided the participant to remove the VR headset and gave the instruction to complete the post-questionnaire. The procedure was concluded with a debriefing containing about the study. Participants were thanked and dismissed from the lab. The whole experimental procedure took about 30 min.

## 4 Experimental Results

In this section, the results of the experimental study are presented using the hypotheses as a structuring element. For the data processing and analysis, the software Statistical Product and Service Solutions (SPSS) in version 22 from IBM was used.

### 4.1 H1: Participants Produce More Pin-Back Buttons in the Augmented Condition Compared to the Non-Augmented Condition

**FIGURE 8:**
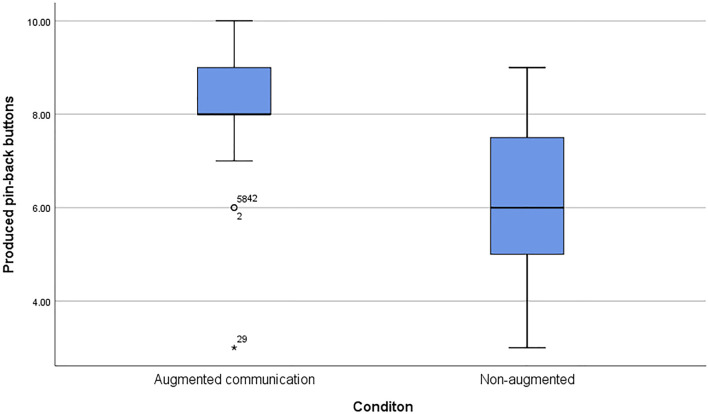
The chart presents the average number of pin-back buttons produced by the participants in collaboration with the robot arm. The augmented condition (*M* = 8.20, *SD* = 1.40) resulted in a higher production output than the non-augmented condition (*M* = 6.15, *SD* = 1.53).

To test H1, an ANCOVA was calculated using the experimental condition as an independent and the production output as a dependent variable and the rating of the augmentation channels, prior experience with industrial robots, and technology affinity (TAM2) as the covariates. Supporting H1 results show a statistically significant difference between conditions (*F*(1,75) = 12.63, *p* < 0.01, $\eta_{p}^{2}=.40$ ). In the augmented condition the average production quantity was higher (*M* = 8.2, *SD* = 1.40) than in the non-augmented condition (*M* = 6.15, *SD* = 1.53) (Figure 8). The production output and the assessment of the augmentation channels were found to be moderate correlated (*r*(80) = 0.39, *p* < 0.01).

### 4.2 H2: Participants Collide Less With the Augmented Robot Arm Compared to the Non-augmented Condition

**FIGURE 9:**
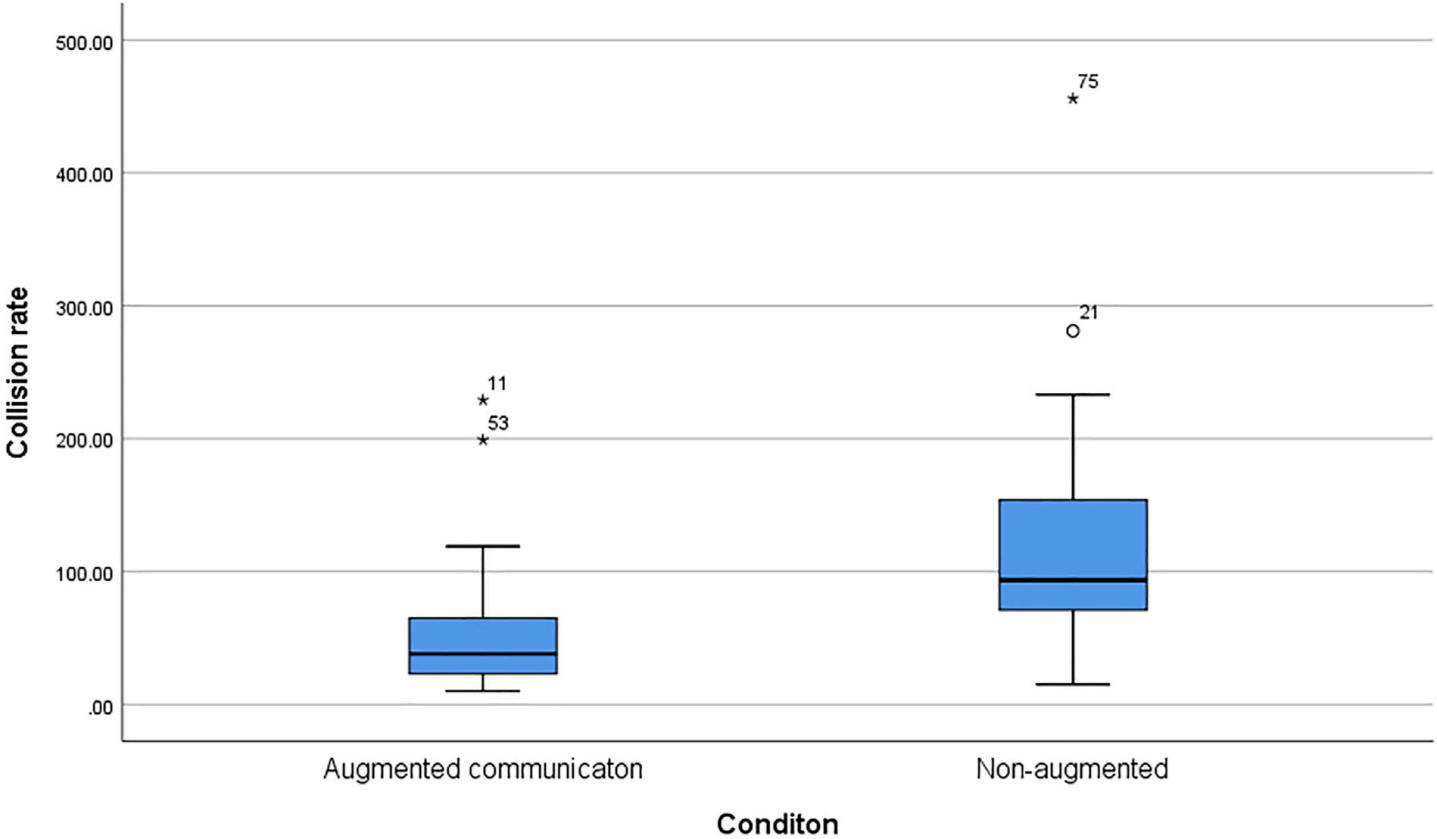
The box plot shows the average collision rate between the robot arm and the participants for the augmented condition (*M* = 53.57, *SD* = 47.40) in comparison to the non-augmented condition (*M* = 118.82, *SD* = 81.49).

H2 was tested by using an ANCOVA with the experimental condition as the independent and the detected collisions as a dependent variable and the assessment of the augmentation channels, prior experience with industrial robots and technology affinity (TAM2) as the covariates. The results revealed a statistically significant difference separating both conditions (*F*(1,75) = 5.93, *p* < 0.01, $\eta_{p}^{2}=.24$ ). The augmented condition on average showed less detected collisions between the participants and the robot arm (*M* = 53.57, *SD* = 47.40) compared to the non-augmented condition (*M* = 118.82, *SD* = 81.49) (Figure 9). Collision rate and assessment of the augmentation channels were found to be correlated (*r*(80) = 0.24, *p* = 0.03) supporting H2.

### 4.3 H3: Perception of Safety is Higher in the Augmented Condition Compared to the Non-Augmented Condition

The third hypothesis was examined by calculating an ANCOVA that contained the experimental condition as the independent variable and the perceived safety rating of the robot arm as the dependent variable with the safety aspects of the workplace as the covariate. The results indicated a statistically significant difference between the two conditions (*F*(1,77) = 5.47, *p* < 0.01, $\eta_{p}^{2}=.12$ ), with the perceived safety rated slightly higher on average in the augmented condition (*M* = 3.33, *SD* = 0.59) compared to the control non-augmented condition (*M* = 3.17, *SD* = 0.58). The results support H3.

### 4.4 Research Question: Does the Time Participants Look at the Text Panel Affect the Productivity and Collision Rate?

Results of the Pearson correlation indicated that there was no statistically significant effect between the time participants looked at the display and the collision rate nor the production output with an average display gaze of 253.02 s (*SD* = 99.61).

## 5 Discussion

With the aim to provide an adaptive and accessible application suitable for HRC experimental studies, we developed a VR sandbox as a modular platform, as described in chapter 3.2. Based on best practices from prior work (Straßmann et al., 2019; Kessler et al., 2020), every mechanic was designed and implemented as a modular component, that can be adjusted, extended, or omitted to fit the current experimental study’s requirements. Apart from a library of assets that can be used to create the virtual environments to emulate industrial workplaces, the VR sandbox provides the tools to enable interactions with a robot as well as with other machinery, inverse kinematics, or the usage of machine learning independent of the robot model to be explored for HRC. This allows the VR sandbox to adapt and replicate a variety of workplace setups involving shared tasks with industrial robots within an authentic and safe environment for HRC research. The usage of simulated industrial environments through augmented and virtual reality is established itself throughout various fields of research (Daling et al., 2020; Dyck et al., 2020; Shahid et al., 2020). However, compared to our VR sandbox, these applications for simulating industrial settings are designed with one specific use case in mind, precluding the usage for an iterative and flexible experimental process (Shu et al., 2018). The usage of a virtual environment comes with certain restrictions, as it is always merely an approximation of the real counterpart. However, real lab-controlled experimental studies similar in scope and objective are also often met with compromise in depicting believable industrial settings (Arntz et al., 2020c). The benefit of the VR sandbox lies in the reduced effort to conduct experimental studies as the functionality can be iterated across different robot representations without starting all over again, compared to experiments conducted within real lab conditions. Another advantage is the simple collection of objective measures that can complement subjective or self-reported qualitative and quantitative measures to explore various research questions regarding HRC.

The goal of this experimental study was to examine the effect of augmented communication on productivity and safety in shared task setups involving the collaboration between humans and autonomous industrial robots. Prior studies conducted within the VR sandbox focusing on subjective measures revealed various benefits of equipping a robot arm with communication channels in HRC setups (Arntz et al., 2020a; Arntz et al., 2020b). Yet one of the key aspects determining the success of HRC remains largely open: the economic point of view, which mainly addresses productivity and safety concerns (Buxbaum et al., 2020). Motivated by this, it is necessary to investigate if the usage of augmented communication can also result in advantages regarding objective measurements such as productivity and safety.

In accordance with the first hypothesis that addresses the number of produced pin-back buttons, participants of the augmented condition generated a higher production quantity compared to the control group. Considering that the assessment of the communicative augmentation strongly correlated with the quantity of produced assets, it can be assumed, that the explanation and guidance provided by the robot arm contributed to participants performing better in terms of productivity. According to human group collaboration research (Shah et al., 2011), the communication channels might contribute to forming distinguished roles within the collaboration process. Participants assigned to the control condition did not receive any guidance and explanation from the robot arm, which required that they fathomed the procedure based on their own mental model (Peltokorpi and Hood, 2019). This probably affected the quantity of produced pin-back buttons, as participants of the control condition, required more time to acclimate to the procedure. Although the task used in this experimental study was fairly simple in execution compared to common industry procedures, the combined objective and subjective results indicate that the augmentation channels can help to support the collaboration process between humans and autonomous robots in terms of production efficiency. While it can be assumed, that industrial employees were familiar with the necessary working steps of their assigned task compared to the inexperienced participants, it can be argued that due to more dynamic production cycles in the future, employees will be exposed to regularly shifting procedures. Communication channels that provide guidance and explanation from the robot, might help to mitigate necessary training time and reduce fear of wrong-doing, therefore contribute to maintaining a high production capacity, consequently support the economic success of the HRC concept. However, since the VR sandbox is capable of recreating a variety of distinct scenarios, it is recommended that future studies extend the complexity of the collaborative task to further investigate the impact of each augmentation channel on people’s productivity.

The second hypothesis stated a reduction in collisions between the robot arm and the participants in the augmented condition. The results support the hypothesis that participants of the augmented condition collided less frequently and that this occurrence correlated with the assessment of the communication channels. Considering that the robot arm’s augmentations enabled it to convey potential hazardous situations through multiple channels, it can be assumed that participants were better suited to recognize these collisions and adapt their behavior to prevent them (Zakka et al., 2019). Although the results show a significant gap between both conditions regarding the collision rate, it can be argued that in a real HRC procedure, the difference would be less significant. The reason for this can be seen in the limitations of the VR technology which currently omits tactile feedback. Although the vibration motors of the Oculus Touch Controller were used to signal a collision, it cannot be ruled out that this stimulus was not correctly interpreted by all participants, thus minor collisions were possibly not noticed by the participants.

The third hypothesis complemented the gathered objective measures of the collision rate with the subjective survey to examine if the potential benefit from the augmentations in safety affected the participants’ perception. The results of the experimental study indicate a contribution of the augmentations of the robot arm towards a stronger perception of safety by the participants. With both the perceived safety of the system and the workplace scored better in the augmented condition, a statistically significant difference could be detected. It can be argued that the information provided by the augmentation channels reduced the uncertainty and therefore contributed towards the impression of a safe system (Seo, 2005; Arntz et al., 2020a). A possibility to strengthen this impression is the inclusion of a backchannel in the communication of the robot arm. Since the perception of safety is influenced by the awareness of an organizational structure within a task, which is formed by exchanging information between those involved in the collaboration (Cigularov et al., 2010). The lack of the ability to respond to the robot i.e., asking to clarify a statement or situation may diminish the impression of group cognition as the criteria for communication exchange is not met (Hart and Staveland, 1988). The presence of the impression of mutual understanding about the current situation within a collaboration setup contributes to the perception of safety. While implementation of the three communication channels that were exclusively one-sided could deliver this understanding for the short and simple task deployed in this setup. A real shared task involving more complex setups might demand a stronger communication exchange (Cigularov et al., 2010). The research question examined the affect of display gaze time on the production output and collision rate. No statistically significant correlation was found. Considering that no dedicated eye-tracking device was used for this measurement, the results might be insufficient regarding the precision of the implementation. It can be argued that the usage of a distinct focal point in the center of each eye respectively detecting an overlap with the virtual display, may not cover any peripheral vision of a VR user. While it can be stated that due to the lenses of the VR headset, which contain only a small focal point in the center for displaying a sharp image to the person wearing the device, usually, the center point is where the user focuses their attention. Therefore vindicating the approach of the implementation. However, it is advised to use proper eye-tracking hardware in future iterations of HRC-related studies involving communication channels to ensure precise data.

### 5.1 Limitations

Limitations include the respective constraints of the VR technology, the study design, and the composition of the sample that is discussed in the following.

While the study used a sophisticated VR headset, the image resolution of the device still diminishes the visual fidelity of the experience. In conjunction with the limited interaction capabilities of the motion-based controller, the usage of VR can only approximate the realism of a shared task study involving a real robot. Influences that are present in real HRC setups, like touching the robot or the components that are part of the collaboration are omitted in VR, resulting in the absence of a sensory channel that might contribute to the assessment of the situation. However, findings from preceding studies suggest that the participants immersed themselves into the experience and even recognized sudden or unexpected movement by the robot arm as threatening (Arntz et al., 2020a; Arntz et al., 2020b), although the VR application posed no real danger. This indicates that the technology is suitable for exploring HRC concepts before they become reality and therefore helps to optimize these workplace setups.

A noteworthy limitation regarding the study design is the usage of the pin-back button press as a shared task. While the usage of a collaborative robot for such a simple task is exaggerated and not appropriate for an industrial context, the relative straightforwardness of the pin-back button machine allowed to establish a comprehensible shared task scenario. Participants independent of prior experience were, therefore, able to execute the procedure and develop a work pace based on the guidance and explanations of the robot arm. Albeit not applicable to complex procedures that are found in industrial manufacturing, the used task allowed to gather insights into the participants’ behavior when exposed to such a scenario. Another limitation in the study design is the short exposure time of the participants with the stimulus material. Considering that industrial employees tasked to collaborate with robots are expected to work with them during prolonged shifts, the dynamic of that relationship that might emerge in this time frame cannot be emulated by the 10 min that were applied in this study. While similar HRC studies are conducted with comparable exposure times for the participants, it is advised to investigate possible deviations from the hereby gathered results in long term studies.

Further worthy of mentioning is the composition of the participants. The sample consisted predominantly of students associated with the field of computer science and engineering. Thus, the gathered results do not apply to the general population and in particular to experienced industrial workers. However, because the presented scenario involving AI-enhanced autonomous robots deployed for collaboration can be anticipated for the future, the usage of students that provide the forthcoming workforce can be argued as appropriate.

An additional limitation in this experimental study is the moderate reliability of the perceived safety scale used for H3 (described in Section 3.3). Although a Cronbach’s alpha value below 0.7 can emerge due to the small number of items used in the scale (Cortina, 1993), further revisions and validations of this scale are required for expanded HRC experimental studies.

## 6 Conclusion

The concept of complementing the individual skills of human employees with the advantages of robots will become ever so important in industries with increasing competitiveness and dynamic production cycles. However, current implementations of shared workspaces between humans and robots are restricted by necessary safety precautions that limit the areas of application where the combined work of robots and humans can create an economic benefit. Augmenting autonomous robots in shared task environments with communication channels shows promise in enhancing production quantity, reducing collision risk, and perceived safety. These factors play a significant role in establishing HRC in the industry, as only an economical and safe implementation of the concept convinces industry decision-makers to adopt this approach. The results of this study indicate that these augmentations that contribute to actual safety by reducing collisions between the robot and the human collaboration partner, also increase the perceived safety of the system. Nonetheless, the tendency for augmentation for autonomous acting robots to award several advantages to the collaboration process, implicates that HRC-related research and the industry should examine different approaches on how to integrate communication-based augmentation into these work scenarios for upcoming production processes. To cover this subject, the presented virtual reality sandbox application provides the first step for a flexible tool to investigate potential solutions for these essential questions for HRC.

## Data Availability

The raw data supporting the conclusion of this article will be made available by the authors, without undue reservation.
